# How to Be Healthy

**Published:** 1855-01

**Authors:** 


### HOW TO BE HEALTHY.

IT is well said, by one who had thoroughly studied the subject, that the highest ambition of an ancient Greek was to be healthy, beautiful and rich. We cannot help thinking, says the Philadelphia Bulletin, that the Athenians, in this respect were wiser than ourselves. Much as we boast of wonderful intelligence, we have not yet practically attained a method of life so comprehensive as that pursued, not only by philosophers, but the men of fashion about in Africa and Poloponesus. They placed health first, and money-making last, while we reverse the order. Yet they were pagans, and we Christians. Surely we should cry "sahmeh" to ourselves.

In reality, the principal objects sought by the ancient Greek, health and beauty, were but one and the same. For beauty cannot exist without health. The man who is constantly confined to the counting desk soon acquires an habitual stoop; the one who devotes his whole soul to money-making becomes wrinkled before his time. On the contrary, he who indulges in proper exercise and recreation, as, for example a well-to-do farmer in healthy districts, carries an erect frame to the verge of seventy, and has a ruddy cheek even when an octogenarian. The first, by neglecting the laws of nature, not only destroys his own manly bearing, "but transmits a puny form and weakly constitution to his children." The last perpetuates a race of hardy sons and majestic daughters.

There is only one way to preserve his health, and that is to live moderately, take proper exercise, and be in the fresh air as much as possible. The man who is always shut up in a close room, whether the apartment be a minister's study, a lawyer's office, a professor's laboratory, or merchant's gas-light store, is defying nature, and must sooner or later pay the penalty. If his avocation renders such confinement necessary during a portion of the year, he can avoid a premature breaking down of the constitution only by taking exercise during the long vacations of the summer and winter months. The waste of stamina must be restored by frequent and full draughts of mountain and sea beach air, by the pursuits of the sportsman, by travel, or other similar means. Every man who has felt the recuperative effects of a month of relaxation, knows from his own experience how genial its influence is; how it sends him back to business with a new flow of spirits; how it almost recreates him, so to speak. Between the lad brought up to physical exercises in the invigorating open air, and one kept constantly at school or in the factory, there is an abyss of difference, which becomes more perceptible every year, as manhood approaches, the one expanded into stalwart, full-chested health, while the other is never more that a half-completed man.

The advantages of exercise are as great in females also; all that we have said about preserving health in man is as true in the opposite sex. But this is not the whole. The true foundation of beauty in woman is exercise in fresh air. No cosmetics are equal to these. The famous Diana of Poicitiers, who maintained her loveliness until she was near sixty, owed this extraordinary result, in her own opinion, to her daily bath, early rising, and her exercise in the saddle. English ladies of rank are celebrated, the world over, for their splendid persons and brilliant complexions, and they are proverbial for their attention to walking and riding, and the hours spent daily out of doors. The sallow cheeks, stooping figures, susceptibility of cold, and almost constant ill-health which prevail among the American wives and daughters generally, are to be attributed almost entirely to their sedantry life, and to the infirmity caused by the same life on the part of their parent. A woman can no more become beautiful, in the true sense of the term, or remain so, without healthful exercise in the open air, than a plant can thrive without light. If we put the latter into a cellar, it dies outright, or refuses to bloom. Shall we wilt our sisters, wives or daughters, by a similar deprivation of what is as necessary to their harmonious development?

In another aspect, the care of health is a more important thing than is usually supposed. There is no doubt that, as between city and country, the population of the former suffers most from want of exercise and fresh air, and that consequently the stamina, so to speak, of a city population is inferior to that of a rural one. It is even said that in some cities, Paris for instance, few strict town-bred families last over a century, and that if the population was not continually recruited from the country, it would die out. It is an equally striking fact, and one that lies within the observation of all of us, that the most energetic merchants generally, in New York, Boston and Philadelphia, have been originally lads from the rural towns or counties, whose well-balanced, vigorous, enterprising minds enabled them to endure an amount of fatigue which the average of their city-competitors could not rival.

The public weal therefore, as well as the happiness of the individual, is concerned in this question of health. Yet we Americans almost ignore it, and practically neglect it entirely. The old Greeks had their gymnasiums for physical exercise, which were as much State institutions as common schools are now. Were not the Greeks wiser, after all, than we are, at least in this particular!—*S. C. Adv.*

